# Bronchoscopic debridement and antifungal therapy as an effective non-surgical approach to concomitant aspergilloma and ABPA: A case report

**DOI:** 10.1177/17534666261480895

**Published:** 2026-08-19

**Authors:** Sarah K. Yeo, Christina S. Thornton, Elaine Dumoulin

**Affiliations:** 1 Department of Medicine, Cumming School of Medicine, 70401 University of Calgary, Calgary, AB, Canada; 2 Department of Microbiology, Immunology and Infectious Diseases, University of Calgary, Calgary, AB, Canada

**Keywords:** allergic bronchopulmonary aspergillosis, aspergilloma, pulmonary aspergilloma, bronchoscopic debridement, case report

## Abstract

Pulmonary aspergillosis refers to a spectrum of lung diseases caused by *Aspergillus* species, ranging from allergic manifestations such as allergic bronchopulmonary aspergillosis (ABPA) to chronic and invasive forms, including chronic cavitary pulmonary aspergillosis (CCPA) and aspergilloma. The coexistence of ABPA and aspergilloma is uncommon and poses unique therapeutic challenges, particularly in those unsuitable for surgical intervention. We present the case of a 26-year-old female with CCPA and concomitant ABPA. Despite prolonged antifungal therapy, imaging revealed a persistent left upper lobe aspergilloma accompanied by elevated total and *Aspergillus*-specific IgE levels. She subsequently underwent bronchoscopic debridement with peri-procedural itraconazole. Following the procedure, she demonstrated significant clinical and immunologic improvement, with more than a 20% reduction in IgE levels and normalization of pulmonary function, which had previously shown a restrictive pattern. This case highlights that a multimodal, minimally invasive approach integrating bronchoscopic debridement with peri-procedural antifungal therapy, offers a viable and effective alternative for non-surgical candidates, promoting both functional and immunologic recovery.

## Plain language summary

Pulmonary aspergillosis is a group of lung conditions caused by a common fungus called Aspergillus. These conditions can range from allergic reactions in the lungs to more long-lasting infections that form fungal balls (aspergillomas). Having both an allergic form and a chronic fungal infection at the same time is uncommon and can be difficult to treat, especially when surgery is not an option. We describe a 26- year-old woman who had both conditions. Even after long-term antifungal medication, a fungal mass remained in her lung and blood tests showed ongoing allergic inflammation. She was treated using a minimally invasive procedure, where the fungal material was removed through a bronchoscope (a camera passed into the lungs), along with antifungal medication around the time of the procedure.After treatment, her symptoms improved, her lung function returned to normal, and markers of allergy in her blood dropped significantly. This case shows that a combined, non-surgical approach can be an effective option for selected patients, leading to improvement in both breathing and immune response.

## Background

Pulmonary aspergillosis comprises a broad spectrum of lung diseases caused by *Aspergillus* species, ranging from hypersensitivity reactions such as allergic bronchopulmonary aspergillosis (ABPA) to invasive pulmonary infections. The clinical manifestation and severity of disease are largely determined by the host’s immune status and underlying structural lung abnormalities.^1^ Aspergillomas develop when *Aspergillus spp* colonize pre-existing pulmonary cavities and are associated with a high mortality rate if left untreated.^2^ The co-existence of ABPA and aspergilloma have infrequently been reported and poses unique therapeutic challenges and is associated with severe disease and frequent relapses.^3,4^ While surgical resection is considered the standard treatment for aspergillomas in symptomatic patients, it is associated with considerable mortality and morbidity.^2^ Furthermore, management becomes particularly complex when aspergillomas occur concurrently with ABPA, as optimal therapeutic strategies are not well defined.^3^ Many patients, particularly those with extensive disease or compromised pulmonary function, may be unsuitable surgical candidates, highlighting the need for non-surgical alternatives.^5–8^ In this report, we present the case of a young woman with both ABPA and aspergilloma successfully managed through a multimodal therapeutic approach combining antifungal treatment with bronchoscopic resection.

## Case presentation

### Initial presentation

We describe the case of a 26-year-old woman with a prior imaging-based diagnosis of pulmonary aspergillosis, initially identified in 2016 during immigration screening when chest radiographs revealed bilateral upper-lobe cavitary lesions containing fungal balls. The reporting of this study conforms to the CARE checklist.^9^ Subsequent microbiologic investigations in 2018, including fungal culture, acid-fast bacillus testing, and bronchoalveolar lavage (BAL) galactomannan, were all negative. In 2022, sputum cultures grew *Aspergillus flavus*, and both total IgE and *Aspergillus*-specific IgE levels were elevated. She was treated for chronic cavitary pulmonary aspergillosis with oral itraconazole for six months, resulting in improvement in symptoms though stable mycetoma on imaging. Surgical resection was considered but deemed unsuitable owing to bilateral disease.

**Figure 1.**
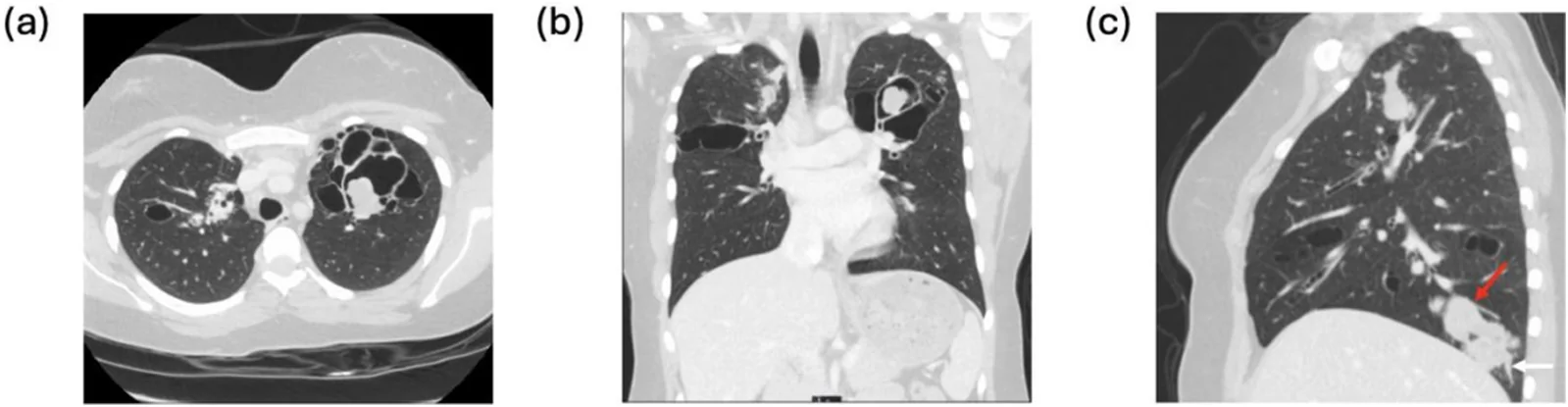
CT findings in a patient with an aspergilloma and concomitant allergic bronchopulmonary aspergillosis. (a) Axial and (b) coronal reconstruction showing an aspergilloma within a cystic cavity in the left upper lobe, (c) Sagittal view demonstrating hyperattenuating mucoid impaction within a dilated bronchus in the right lower lobe (red arrow), with adjacent mild tree-in-bud nodularity (white arrow).

At time of referral to the clinic in 2024, the patient reported recurrent symptoms characterized by persistent scant hemoptysis and a chronic productive cough. Computed tomography of the chest revealed a persistent left upper lobe cavitary lesion containing an aspergilloma, accompanied by upper-lobe–predominant saccular bronchiectasis (Figure 1). Serologic testing revealed peripheral eosinophilia (1.6 × 10^9^/L) and markedly elevated total IgE (14,942 IU/mL) and *Aspergillus*-specific IgE (28.3 kU/L), consistent with concomitant allergic bronchopulmonary aspergillosis (ABPA). Serial pulmonary function tests obtained between 2022 and 2024 showed a stable restrictive ventilatory defect with preserved diffusing capacity and no evidence of obstruction (Table 1).

**Table 1.** Serial pulmonary function testing from 2022 to 2025 demonstrating post-intervention improvement in spirometry following combined medical management and bronchoscopic debridement (completed March 2025).

Date	FEV_1_	FVC	TLC	DLCO (mL/min/mmHg)
October 25, 2022	2.23L (69%)	2.77L (75%)	3.55L (63%)	22.4 (79%)
September 17, 2024	2.21L (62%)	2.93L (70%)	4.33L (74%)	23.8 (98%)
October 22, 2024	2.72L (77%)	3.23L (78%)	--	--
April 22, 2025	2.99L (95%).	3.46L (97%)	--	--
August 5, 2025	2.96L (89%)	3.45L (89%)	--	--

### Therapeutic intervention

**Figure 2.**
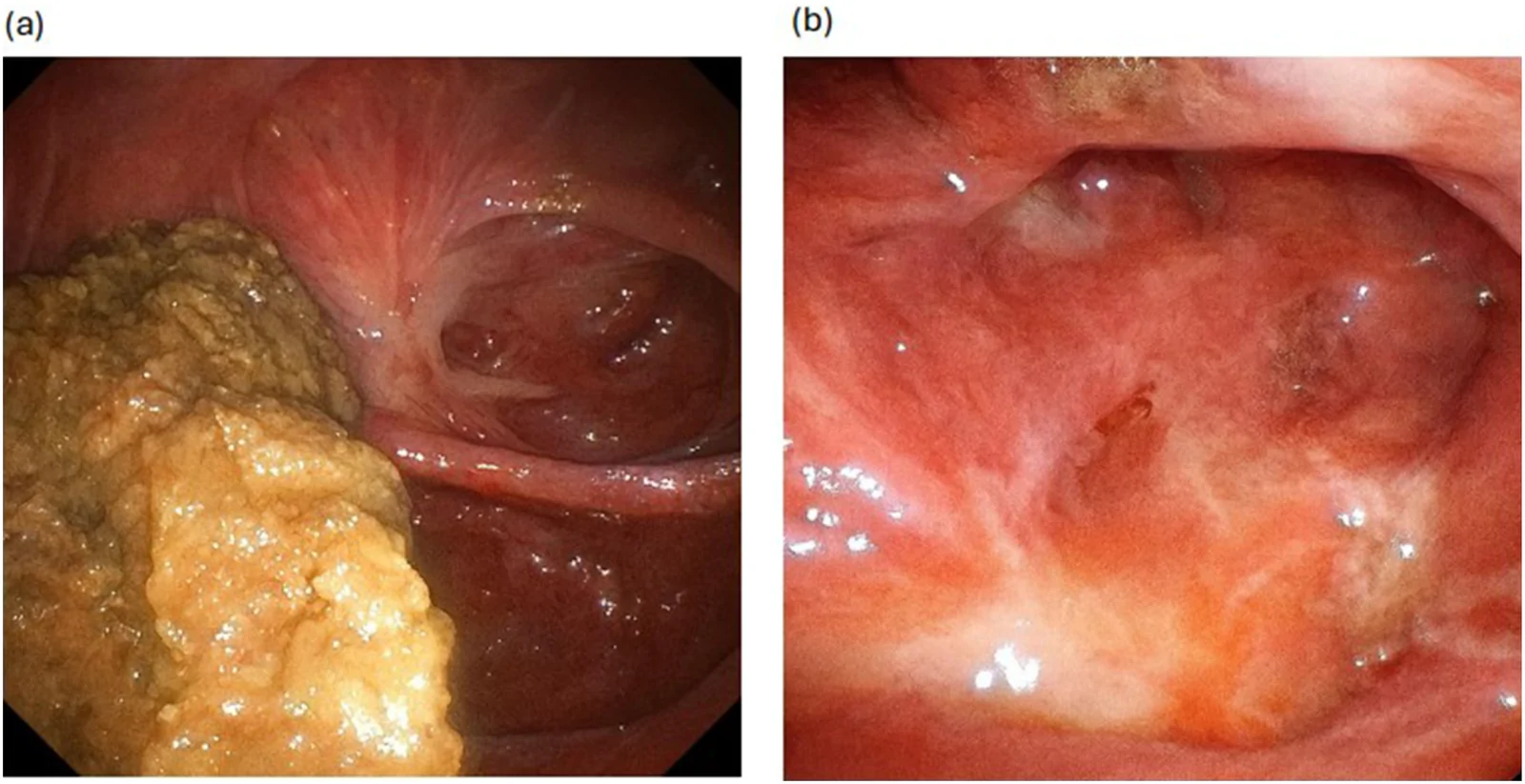
Bronchoscopic images of the left upper lobe before and after debridement. (a) Left upper lobe bronchus demonstrating an aspergilloma. (b) Post-bronchoscopic debridement view of the same segment showing resolution of the aspergilloma with healthy underlying mucosa.

She was subsequently referred to the University of Calgary Aspergilloma Program, where she completed a 16-week course of itraconazole. Following antifungal therapy, she underwent bronchoscopic intervention under general anesthesia using a standard endotracheal tube (size 7.0). An ultrathin bronchoscope (Olympus MP190, outer diameter 3.0mm - working channel 1.7 mm) was utilized to facilitate navigation to distal bronchial segments, as its smaller diameter allows improved access to peripheral airways in the setting of bronchiectasis with associated architectural distortion. (Figure 2(a)). The left upper lobe cavity was accessed directly, allowing visualization of the intracavitary aspergilloma measuring 23.9 x 15.4 x 21.4 mm. (Figure 2(a)). The fungal mass was debrided using a 12 mm Zero Tip nitinol stone retrieval basket (Boston Scientific), which provided improved maneuverability compared to a 16 mm device within the cavity and allowed better control during debridement of the mycetoma. Multiple passes were required to adequately debulk the aspergilloma, with fragments subsequently removed via suction through the working channel of the bronchoscope until the cavity appeared free of fungal debris (Figure 2(b)). Evaluation of the contralateral right upper lobe revealed a residual cavity without evidence of active mycetoma. Rigid bronchoscopy was not required. Tranexamic acid, epinephrine, and a bronchial blocker were available in the operating room but were not utilized during the procedure. The patient was monitored in the recovery unit of the operating room for one hour, followed by an additional two hours in day surgery unit, and was discharged home the same day after being weaned off of supplemental oxygen. There were no complications following the procedure.

### Post-procedural course and outcome

Post-procedurally, she received a short course of prednisone (50 mg daily for five days) and amoxicillin–clavulanate (10 days) to prevent an ABPA flare, followed by an additional three-month course of itraconazole. The combined medical and bronchoscopic regimen resulted in significant clinical improvement, with total serum IgE decreasing from 14,942 to 4,065 IU/mL and peripheral eosinophil counts falling from 1.6 × 10^9^/L to 0.8 × 10^9^/L one-month post-debridement. Repeat pulmonary function testing performed after bronchoscopic debridement in March 2025 demonstrated normalization of lung volumes, with improvements in FEV_1_ and FVC on the April and August 2025 spirometry, indicating recovery from the previously observed restrictive ventilatory pattern and overall improvement in respiratory function (Table 1).

## Discussion

Current clinical guidelines provide limited direction on the optimal management of aspergillomas in patients who are unsuitable for surgery, particularly when complicated by concomitant ABPA. In recent years, less invasive alternatives such as bronchoscopic interventions have been deemed as viable options and offers the advantage of targeted mechanical debridement with reduced procedural risk and preservation of lung parenchyma compared to surgery.

Our centre has previously described a multimodal management protocol for pulmonary mycetoma that has been successful in more than 30 patients at the University of Calgary.^5–8^ Several factors are important for the safe and effective application of this approach, including appropriate patient selection and procedural feasibility. Ideal candidates should be clinically stable, able to tolerate rigid or flexible bronchoscopy, and have a patent airway providing access to a well-defined cavity with minimal surrounding parenchymal involvement. Pre-operative antifungal therapy for three months is used to decrease fungal burden and reduce the risk of contaminating adjacent healthy lung tissue, while post-procedural antifungal therapy, typically continued for one to three months, minimizes relapse risk. However, the optimal duration and intensity of antifungal therapy pre- and post-procedurally remains uncertain and current evidence is limited to small case series and single-center experiences, with substantial variability in treatment duration, antifungal selection, and follow-up protocols.^10^ Further studies are needed to establish standardized post-procedural antifungal regimens and to clarify their impact on long-term recurrence rates, immune modulation, and pulmonary function recovery.

The coexistence of ABPA, as defined by the ISHAM 2024 diagnostic criteria,^11^ and aspergilloma is uncommon and presents significant clinical challenges due to the complexity of management strategies required for each condition. According to the revised ISHAM 2024 guidelines, oral prednisolone or itraconazole monotherapy remain central in controlling the immunologic inflammation of ABPA.^11^ However, management becomes more challenging in the presence of an aspergilloma, where immunosuppression may promote fungal proliferation within cavitary lesions.^12,13^ In the present case, systemic corticosteroids were initially deferred because there was no clear evidence of an acute ABPA exacerbation and concern existed regarding worsening fungal burden and cavitary progression. Antifungal agents may serve as steroid-sparing options, though their efficacy is limited in patients with aspergillomas due to poor drug penetration into the cavity.^7^ Despite these limitations, reports have described clinical improvement, reductions in IgE levels, and partial radiographic resolution of mycetoma following corticosteroid therapy or in combination with anti-fungal agents in cases of coexistent ABPA and aspergilloma.^3,12–15^ Nevertheless, disease recurrence has frequently been reported despite initial response with medical therapy.^13,16,17^ Surgical resection has also been described, particularly in patients with significant hemoptysis or symptoms refractory to medical management.^16,18^ However, postoperative recurrence of ABPA remains a challenge, as surgery does not address the underlying immunologic hypersensitivity. Relapse despite complete resection is commonly described post-operatively, suggesting that maintenance corticosteroids or antifungal therapy may be required to control the allergic component and prevent recurrence.^14,16,18,19^ Adjunctive antifungal agents such as itraconazole and voriconazole have shown benefit in this setting, with peri-operative use ranging from six weeks to 18 months which demonstrated reductions in IgE levels and symptomatic improvement.^16,17,20^

This case highlights that a combined medical and bronchoscopic approach, as previously described by our centre, remains effective even in the setting of an aspergilloma complicated by concomitant ABPA, achieving meaningful clinical, radiologic, and immunologic improvement. The successful reduction in fungal burden through targeted endoscopic debridement, coupled with antifungal therapy and immunomodulatory support, underscores the feasibility of this minimally invasive strategy as an alternative to surgical resection in appropriately selected patients.

## Conclusion

Although rare, our case highlights a practical and well-tolerated approach to the management of concomitant ABPA and aspergillomas in non-surgical candidates using pre and post procedural itraconazole in combination with bronchoscopic excision. These findings suggest that this strategy may serve as viable alternative to surgical intervention.

## Data Availability

The datasets used and/or analyzed in the current study are available from the corresponding author upon request.*
